# Special Considerations in the Care of Women With Advanced Heart Failure

**DOI:** 10.3389/fcvm.2022.890108

**Published:** 2022-07-11

**Authors:** Imo A. Ebong, Ersilia M. DeFilippis, Eman A. Hamad, Eileen M. Hsich, Varinder K. Randhawa, Filio Billia, Mahwash Kassi, Anju Bhardwaj, Mirnela Byku, Mrudala R. Munagala, Roopa A. Rao, Amy E. Hackmann, Claudia G. Gidea, Teresa DeMarco, Shelley A. Hall

**Affiliations:** ^1^Division of Cardiovascular Medicine, University of California, Davis, Sacramento, CA, United States; ^2^Division of Cardiovascular Medicine, Columbia University Irving Medical Center, New York, NY, United States; ^3^Division of Cardiovascular Medicine, Temple University Hospital, Philadelphia, PA, United States; ^4^Cleveland Clinic Lerner College of Medicine of Case Western Reserve University School of Medicine, Cleveland, OH, United States; ^5^Department of Cardiovascular Medicine, Kaufman Center for Heart Failure and Recovery, Heart, Vascular and Thoracic Institute, Cleveland Clinic, Cleveland, OH, United States; ^6^Department of Cardiology, Toronto General Hospital, Toronto, ON, Canada; ^7^Houston Methodist Debakey Heart & Vascular Center, Houston, TX, United States; ^8^Department of Advanced Cardiopulmonary Therapies and Transplantation, McGovern Medical School, University of Texas-Houston, Houston, TX, United States; ^9^Division of Cardiology, Department of Medicine, University of North Carolina at Chapel Hill, Chapel Hill, NC, United States; ^10^Department of Cardiology, Miami Transplant Institute, University of Miami Miller School of Medicine/Jackson Memorial Hospital, University of Miami, Miami, FL, United States; ^11^Division of Cardiology, Krannert Institute of Cardiology at Indiana University School of Medicine, Indianapolis, IN, United States; ^12^Department of Cardiovascular and Thoracic Surgery, University of Texas SouthWestern Medical Center, Dallas, TX, United States; ^13^Leon H. Charney Division of Cardiology, Department of Medicine, New York University Langone Health, New York, NY, United States; ^14^Division of Cardiology, University of California, San Francisco, San Francisco, CA, United States; ^15^Division of Cardiology, Baylor University Medical Center, Dallas, TX, United States

**Keywords:** advanced heart failure, heart transplant, ventricular assist device, women, advanced therapies (ATs)

## Abstract

Advanced heart failure (AHF) is associated with increased morbidity and mortality, and greater healthcare utilization. Recognition requires a thorough clinical assessment and appropriate risk stratification. There are persisting inequities in the allocation of AHF therapies. Women are less likely to be referred for evaluation of candidacy for heart transplantation or left ventricular assist device despite facing a higher risk of AHF-related mortality. Sex-specific risk factors influence progression to advanced disease and should be considered when evaluating women for advanced therapies. The purpose of this review is to discuss the role of sex hormones on the pathophysiology of AHF, describe the clinical presentation, diagnostic evaluation and definitive therapies of AHF in women with special attention to pregnancy, lactation, contraception and menopause. Future studies are needed to address areas of equipoise in the care of women with AHF.

## Introduction

Heart failure (HF) mortality is greater among women than men at all ages in US (1). In 2018, HF was the implicated cause of 83,616 deaths (38,487 males and 45,129 females) (1). Approximately 300,000 HF patients in the US currently have advanced HF (AHF), and an additional 5% will progress to advanced disease each year (2). Estimates of the prevalence of AHF varies from 5 to 25% between studies (2), and the exact proportion of women of reproductive age who have AHF is unknown. AHF is associated with high morbidity and mortality, and huge healthcare-related costs, especially in the last year of life (3, 4). The 1-year mortality estimated by HF survival models is >20%−25% (4). In crude analyses, the heart transplantation (HT) rate for women and men were 0.789/100,000 and 2.33/100,000 respectively each year (5). Using data from the United Network for Organ Sharing and Centers for Disease Control and Prevention the HT to HF mortality ratio was 0.263 for women and 0.424 for men, supporting reports that irrespective of disease severity, less women than men receive a HT (5).

AHF is defined as the presence of progressive and/or persistent severe signs and symptoms of HF despite optimized medical, surgical, and device therapy (3, 6). While sex-related differences in epidemiology, risk factors, pathophysiology, response to therapies and outcomes in HF have been reported (7, 8), the unique characteristic of AHF in women and the influence of sex hormones has not been extensively described. Additionally, there is minimal data on female sex-specific cardiovascular risk factors in the evidence that directs current HF practice guidelines (9). When considering HF subtypes, women have a higher prevalence of HF with preserved ejection fraction (HFpEF) than men, which is rarely an indication for HT or left ventricular assist device implantation (LVAD) implantation (10). Among those with AHF and recurrent hospital admissions, women have a similar prevalence of HFpEF and HF with reduced ejection fraction (HFrEF), while most men with recurrent hospital admissions have HFrEF (5). Age-adjusted case fatality rates from the Atherosclerosis Risk in Communities study showed that HFrEF contributes to more mortality than HFpEF in women (11).

The management of AHF in women is complicated because detection by patients, their families and providers is often delayed (3). Recognizing AHF in women requires a thorough clinical assessment and risk stratification (4). Little is known about the impact of sex on HT allocation or the potential effect of gender bias on the decision-making process for other AHF therapies (12). Data from the organ procurement and transplantation network shows less women than men on the HT waiting list or receiving a HT, and this proportion further declines with age (Table 1). Younger women (<50 years) comprised only 9.6% of the total number of patients on the waitlist and 10.17% of total number who received a HT. The purpose of this review is to discuss the role of sex hormones on the pathophysiology of AHF, describe the clinical presentation, diagnostic evaluation and definitive therapies of AHF in women with specific attention to pregnancy, lactation, contraception and menopause, while considering barriers to treatment.

**Table 1:** Heart transplant waiting list and allocation according to sex and age-groups.

		**Waiting list^a^**		**Number transplanted^b^**	
	**Sex**	**Male**	**Female**	**Male**	**Female**
**Age-group**					
18–34 years		239 (0.67)	120 (0.33)	4,790 (0.62)	2,964 (0.38)
35–49 years		489 (0.74)	168 (0.26)	12,297 (0.73)	4,499 (0.27)
50–64 years		1,126 (0.79)	293 (0.21)	30,321 (0.78)	8,480 (0.22)
>65 years		464 (0.83)	98 (0.17)	8,253 (0.82)	1,766 (0.18)
Total		2,318 (0.77)	679 (0.23)	55,661 (0.76)	17,709 (0.24)

*Values are number (%) for the respective column*.

a *Based on organ procurement and transplantation network data as of June 1, 2022*.

b *US transplants performed: January 1, 1988–April 30, 2022*.

*Source: Organ procurement and transplantation network. Heart resources and services administration, US Department of health and human services. https://optn.transplant.hrsa.gov/data/*.

## Pathophysiology Of Advanced Heart Failure In Women: The Role Of Sex Hormones

**Figure 1:**
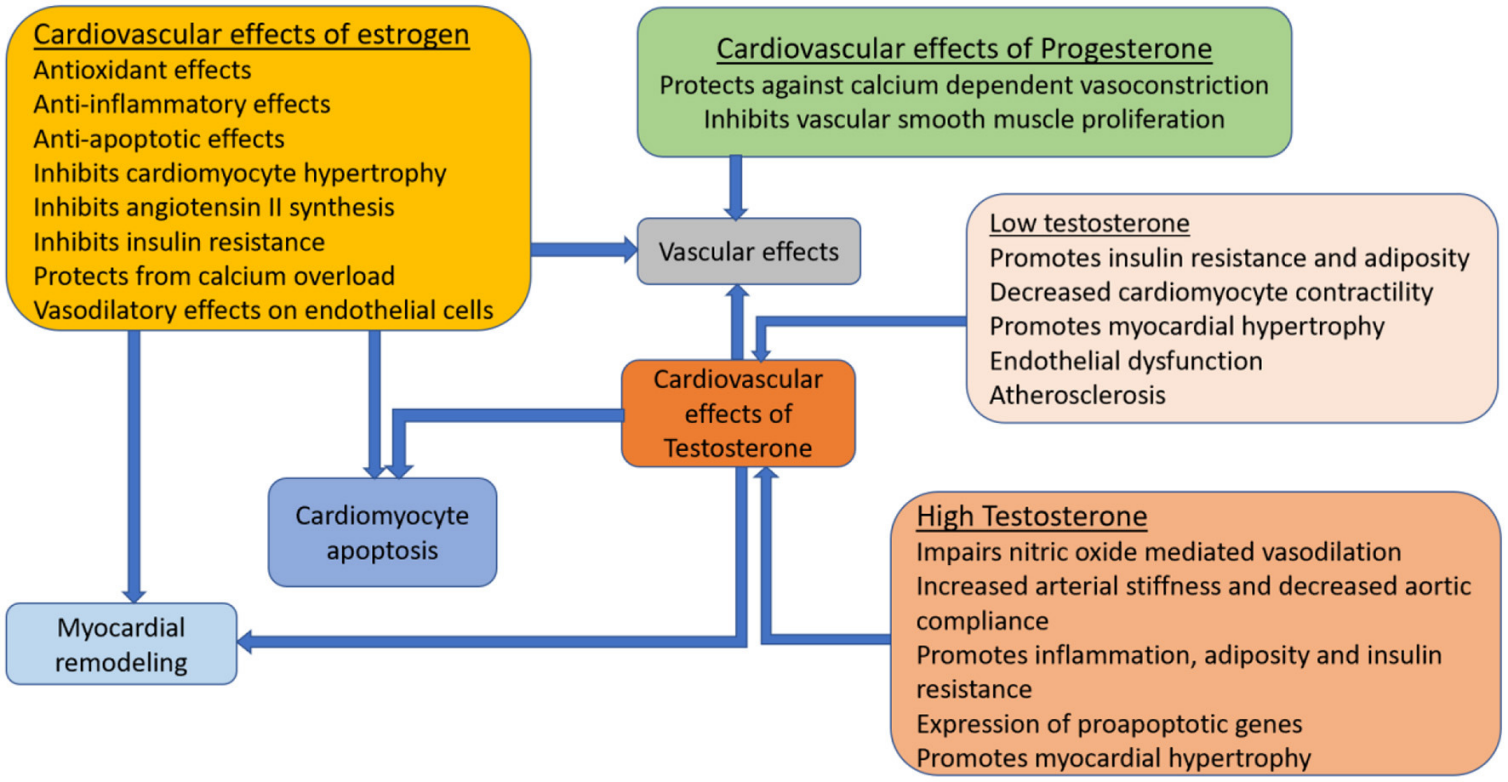
Influence of sex-steroid hormones on the cardiovascular system. Cardiomyocytes and vasculature have receptors for major female sex-steroid hormones; estrogen, progesterone and testosterone. These sex-steroid hormones influence heart failure progression through cellular mechanistic processes that have direct and indirect effects on the myocardium and vascular tone.

The pathophysiological mechanisms underlying HF involve the activation of structural, neurohumoral, cellular, and molecular pathways in response to myocardial injury in an attempt to maintain homeostasis (13). Sex hormones including estrogen, progesterone and testosterone modify some of the pathophysiological processes that promote HF progression in women (Figure 1). Estrogen modulates the expression of proteins that regulate vascular tone and response to myocardial injury, and influences ventricular contractile function, endothelial calcium metabolism, coronary calcification, coagulation and fibrinolysis, insulin resistance, inflammation and lipid oxidation (14). Through these actions, it limits cardiac remodeling and attenuates myocardial hypertrophy (15). Progesterone affects vascular tone by modulating calcium channel activity, inhibiting vascular smooth muscle proliferation and migration, and worsening the response to vascular injury (16, 17).

Androgen excess is associated with greater risk of heart disease in women, due to its adverse effects on the vasculature, lipoprotein levels and adiposity (16, 18, 19). Low androgen levels are also associated with atherosclerosis and coronary artery disease (19). Elevated testosterone promotes cardiac remodeling by causing myocardial hypertrophy, modulating the autonomic nervous system and regulating excitation contraction coupling through its effects on intracellular calcium levels (16). Physiological levels of testosterone improves endothelial function, peripheral vascular resistance and vasomotor tone, and its effects on the cardiovascular system depends on circulating estrogen levels and the peripheral conversion of testosterone to estradiol (19).

The effects of female sex hormones on HF pathophysiology is a continuum, that persists with progression to advanced disease, such that AHF is not an end-state but a dynamic condition where numerous mechanical, molecular, immunologic, ischemic, proarrhythmic, vascular, and musculoskeletal forces contribute to symptoms and continuing deterioration (20). There is increasing inability to meet the metabolic demands of end-organs and skeletal muscle, renal and hepatic dysfunction, and reduction in exercise capacity, cachexia, and fatigue (3). Estrogen, progesterone and testosterone receptors continue to activate cellular mechanistic cascades that modulate inflammation, apoptosis, vascular abnormalities and myocardial remodeling in response to worsening pathologic conditions in AHF (16).

## Clinical Presentation Of Advanced Heart Failure In Women

There is no specific event that marks the progression to AHF, instead a pattern of clinical findings may be the optimal indicator (3). AHF is characterized by recurrent hospitalizations, escalation of diuretics, intolerance or dose-reduction of guideline directed medical therapies, development of end-organ dysfunction, cardiac cachexia, and refractory arrhythmias with or without device shocks (3). Recurrent hospitalization is a strong indicator of progressive decompensation as HF approaches its late stages (4). Failure to respond to conventional therapies, another manifestation of disease progression, presents with persistent functional impairment which can be nonspecific in elderly patients (3) and women. The absence of a sex-oriented assessment of disease severity could make the identification of AHF among women a persistent challenge for clinicians (21). However, the use of gender-specific risk prediction models did not improve the accuracy of predicting mortality risk in decompensated HF (22).

Women with AHF experience higher symptom burden, poor coping strategies, and greater prevalence of depression and social isolation than their male counterparts (23). As the disease progresses, its impact on functional status and quality of life is more debilitating among women, not just from HF alone, but the greater burden of comorbidities and older age of female HF patients (21). Frailty and cachexia, common features of AHF (4, 24), are more frequently seen in women (24). Physical frailty is characterized by worse symptom characteristics in women, and worse body composition characteristics in men (25). However, current frailty assessment tools are not sex-specific, and future research is needed to identify the ideal index for frailty assessment in women, and sex differences in reversibility of frailty with HF therapies (25). Women are also admitted less frequently than men for acute decompensated HF (1) which may lead to delayed recognition of advanced disease. Consequently, the gender distribution of patients referred for AHF therapies likely does not represent the actual proportions of patients with AHF.

## Diagnostic Evaluation Of Women With Advanced Heart Failure

The initial evaluation of AHF should be focused on excluding reversible causes and ensuring adequate treatment with maximally tolerated guideline directed medical therapies (26). For women with persisting features of hemodynamic instability or systemic hypoperfusion, with or without end-organ dysfunction, the evaluation process becomes more structured to establish candidacy for advanced therapies by identifying contraindications to heart transplantation (HT) or left ventricular assist device (LVAD) implantation (27). The evaluation process is both comprehensive and center-specific (26) and eligibility determined after review by a multidisciplinary selection committee.

Cardiopulmonary exercise testing provides objective information about cardiovascular reserve and prognosis (6). In women, a peak oxygen consumption ≤ 50% of expected is a recommended parameter for consideration for advanced therapies (28), since women exhibit better survival than men for any given peak oxygen consumption value (29). Invasive hemodynamics obtained from right heart catheterization provides information that guides specific pharmacotherapy and durable therapies by enabling precise assessment of filling pressures, pulmonary hypertension, cardiac output, and right ventricular performance (26). Sex differences in hemodynamics have not been systematically explored. However, in the SHOCK registry, cardiac power index, a strong predictor of mortality was significantly lower in women (30). Consequently, physiological differences between men and women must be acknowledged when interpreting functional testing. In the REVIVAL study, the 6-min walk test distance was significantly shorter in women than men by almost 40 m despite similar age and functional class (31). Cardiac biomarkers further improve risk stratification and selection for advanced therapies. Women with decompensated HF have higher natriuretic peptide levels than men for any given LV ejection fraction (32, 33), and natriuretic peptides are stronger predictors of HF-related mortality in women (34). Natriuretic peptides are influenced by adiposity, menopause and sex hormones, such that women have lower levels after the onset of menopause (35). It is unclear if sex-specific cut-offs in biomarker levels should be adopted for AHF prognostication (33).

## Life Sustaining Therapies For Women With Advanced Heart Failure

Gender disparities persist in the utilization of AHF therapies (36). In a multicenter retrospective analysis, 73.4% of referrals evaluating candidacy for advanced therapies were men (37). Women are allocated to less than a third of HT and LVAD in the US (38) and are under-represented in HF clinical trials (5, 7, 10).

### Heart Transplantation

Despite having shorter waitlist times and greater HF-related mortality, women are less likely to receive a HT than men (39). Among patients transplanted yearly in US, women received only 26% while men received 74% of donor hearts (39). Gender disparities in HT are a consequence of fewer women being listed for transplant, greater waitlist mortality for women, less aggressive HF treatment in women, and organ allocation factors like allo-sensitization, which limits the availability of potential donors (39, 40).

Among patients who require hemodynamic stabilization prior to HT, women are more frequently bridged with inotropic support and less likely to receive mechanical circulatory support (MCS) as a bridge to transplantation (39). LVAD is an important bridging therapy that maintains cardiac function while awaiting HT, therefore, its underutilization could contribute to increased mortality during the pre-transplant period (40). However, it has also been reported that women who are supported with an LVAD as bridge to transplant have lower chances of HT than men, higher waitlist mortality, increased delisting for worsening clinical status and are less likely to be transplanted urgently (38, 41). Future studies are needed to explore the optimal waitlist strategy for women.

After HT, women tend to have better long-term survival than men, lower risk of coronary allograft vasculopathy and malignancy, but a higher risk of antibody-mediated rejection (39). Sex matching has less impact on early mortality among female transplant recipients, but, survival after 5 years is better among female recipients matched to female donors in comparison to women matched to male donors (8).

### Left Ventricular Assist Devices

Regardless of the indication for implant, there are sex-related disparities in the utilization and outcomes of LVAD as bridge to transplant, bridge to recovery or destination therapy (8). Data from the INTERMACS registry involving 18,868 patients who received their first continuous flow-LVAD between June 2008 and December 2017, showed that women comprised only 21.1% of LVAD recipients mostly for a bridge to transplant indication (42). This disparity may be because women are referred later for advanced therapies (2), when they are no longer candidates for durable LVAD.

Despite mixed evidence, women appear to have similar complication rates as men with use of contemporary LVADs including in-hospital mortality, time to infection, post-operative bleeding, and device malfunction, however, stroke and early right ventricular failure are more common in women (38, 43). Female LVAD patients are at higher risk of both hemorrhagic and ischemic stroke, but the risk of hemorrhagic stroke is greater among women <65 years while ischemic stroke risk is greater among women ≥65 years (39). The factors that underlie gender differences in thromboembolic risk and responses to anticoagulation could similarly explain gender disparities in stroke risk after LVAD implantation and should be explored in future studies. Right ventricular failure is also more common in women than men after LVAD implantation, with some evidence supporting later presentation and higher prevalence of non-ischemic cardiomyopathy as contributing factors (44).

Although many studies show few sex differences with LVAD usage, women with continuous flow-LVADs who were ≤49 years old were at increased risk of mortality in comparison to men of similar age in a study by Gruen et al. using the INTERMACS registry (42). In the same study, women had greater likelihood of adverse events including pump thrombosis, infection, bleeding and stroke (42). In another study, women with ischemic HF etiology had greater LVAD mortality risk than men (45). Other studies limited to continuous flow devices have shown comparable post-LVAD complication rates in both sexes (46). In an analysis of the National Inpatient Database by Ahmed et al. from January 2009 to December 2014 (mainly HeartMate II and HeartWare), there were no significant gender differences in in-hospital mortality or complications after LVAD implantation (47). It is unclear how gender biases in selection arising from differences in clinical severity or psychosocial issues could influence LVAD outcomes. To further address conflicting data, more sex-specific LVAD research is needed, especially limited to contemporary LVADSs like HeartMate 3 which has a lower rate of adverse events (44).

### Temporary Mechanical Circulatory Support

Temporary MCS (TMCS) devices such as intra-aortic balloon pump, micro-axial LVADs, extracorporeal membrane oxygenation and TandemHeart can provide uni- or biventricular support to patients with AHF or cardiogenic shock. Current trends show a decrease in intra-aortic balloon pump use and increases in micro-axial LVADs and extracorporeal membrane oxygenation use in both sexes (48). Despite being sicker at presentation (44), TMCS is underutilized in women (49) and is associated with greater complication rates, including vascular complications that sometimes require surgical interventions (50). Women experience greater mortality from cardiogenic shock than men despite TMCS use (49). In acute myocardial infarction related-cardiogenic shock, women received TMCS support less frequently (48) even though Impella support prior to percutaneous coronary intervention is associated with greater survival benefits in women than men (44). Further research is required to explore sex-based differences, hormonal influences and potential anatomical considerations in TMCS utilization and outcomes.

### Palliative Care and Inotrope Use

Palliative care is an interdisciplinary approach to patient management that focuses on reducing suffering and improving quality of life in serious illness such as AHF, for patients and their caregivers (51). Although current evidence shows an increasing trend in palliative care use in AHF, it remains underutilized in US with an estimated adoption rate of 6.2% in 2017 (52). There are sex disparities in response to palliative care interventions, with women experiencing less improvement in patient-reported outcomes than men (23). This may be because women with AHF have higher levels of distress before their symptoms are acknowledged and managed by their providers (23, 53). Despite excessive mortality associated with their use (2), palliative inotropes (milrinone or dobutamine) improve HF symptoms and decrease hospital admissions (54), making them an option for terminally ill patients who are not candidates for HT or LVAD. When used as a bridging strategy, men are over seven-times less likely to be successfully bridged to HT with long-term milrinone support than women (55). A sex-specific approach to the use of palliative care interventions is necessary to improve outcomes among women with AHF (23).

## Special Considerations

**Figure 2:**
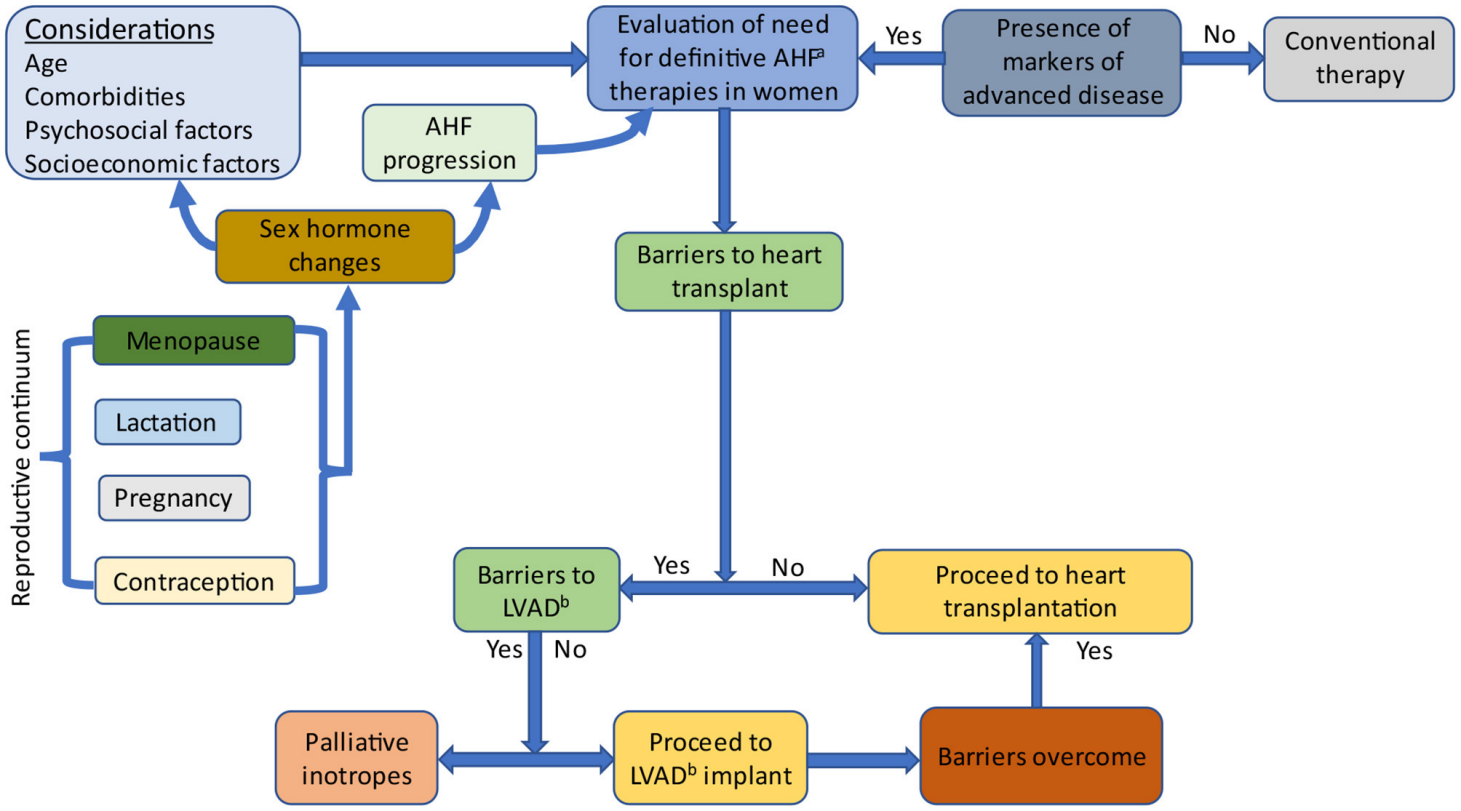
Evaluation of women for advanced heart failure therapies. Sex-specific factors affect hormonal levels and include pregnancy, lactation, contraception and menopause. Sex hormones influence heart failure progression and factors which are considered when evaluating women for advanced heart failure therapies. AHF, Advanced heart failure; LVAD, Left ventricular assist device.

The reproductive continuum spans contraception use, pregnancy, lactation and the menopausal transition, resulting in sex hormonal changes that affect HF development and progression (Figure 2). Irrespective of their life stage, similar considerations should be applied when evaluating women for AHF therapies.

### Pregnancy

The progression of HF during pregnancy varies according to the underlying cardiomyopathy, and may be aggravated by physiological changes experienced during pregnancy (56). HF during pregnancy, although relatively rare (57, 58), is associated with increased risks of maternal and fetal complications (57) and is the most common cause of pregnancy-related death in developed countries (59, 60). Decompensation most commonly occurs during the second or third trimester, or shortly after delivery (56). HF in pregnancy may be due to pre-existing cardiac diseases such as congenital heart disease, non-ischemic cardiomyopathy, valvular disorders, hypertrophic cardiomyopathy or peripartum cardiomyopathy (PPCM) (56, 58). PPCM, an idiopathic cardiomyopathy with LVEF <45% that occurs toward the end of pregnancy or in early months after delivery, abortion or miscarriage, without other known causes of HF, is the most common cause of HF during pregnancy (56, 58, 61, 62). Irrespective of the underlying cardiomyopathy, women with severe functional impairment, moderate to severe LV dysfunction, hemodynamic load such as LV outflow tract obstruction and pulmonary hypertension should be counseled against pursuing pregnancy (59).

Women with AHF who become pregnant should be informed about the risk of deterioration, and therapeutic abortion offered to those with extreme risk in early pregnancy (60, 63). A multidisciplinary management strategy involving high risk obstetrics, neonatology, anesthesiology, HF cardiology, and cardiothoracic surgery should be pursued early in pregnancy (60, 64). The onset of hemodynamic instability and cardiogenic shock with need for inotropic or vasopressor use at any time during gestation should prompt referral to a tertiary center with capabilities for MCS and urgent delivery by cesarean section (63). Vaginal delivery should be considered if the woman is hemodynamically stable (60). However, in emergency situations, advanced therapies and drugs that are not recommended during pregnancy should not be withheld. HT should be considered for patients who fail to recover after delivery despite maximal therapies (63). In PPCM, there is an increased potential for graft failure and death after HT in comparison to other HF etiologies (62), so HT should be reserved for women with refractory severe HF where LVAD is not possible or desirable, due to biventricular failure or severe initial right ventricular dysfunction (61). Women who desire pregnancy after HT should be counseled on the appropriate timing and management of pregnancy, and educated on the increased risk of cardiac allograft rejection and dysfunction, infection, and teratogenicity associated with use of immunosuppressive agents (63, 65). Pregnancy is not recommended in women supported with LVAD (66).

### Lactation/Breastfeeding

Breastfeeding is associated with positive cardiometabolic changes including reduced insulin resistance, lower fasting glucose and blood pressure (67). Therefore, lactation may lower cardiovascular and HF risk. However, it is unclear if the reduction in cardiovascular risk factors could mitigate HF progression or ameliorate advanced disease. In PPCM specifically, prolactin suppression with bromocriptine (a dopamine agonist) was associated with greater LV functional recovery (68). The European Society of Cardiology recommends against breastfeeding when LV function is severely impaired but encourages breast feeding in women with mild systolic dysfunction (69). Guideline directed medical therapy can be used during lactation with careful attention to the safety profile of each medication and its possibility of being secreted in breastmilk (63, 66). The decision to pursue breastfeeding among mothers who are HT recipients should also be individualized and based on a risk-benefit analysis of the potential for immunosuppressive medications to be excreted in breast milk (65).

### Contraception

Providers caring for women with AHF of reproductive age should inquire about contraceptive use, because pregnancy can lead to hemodynamic compromise (63). Contraceptive options include combined hormonal oral contraceptives, progestin-only formulations, intrauterine devices, barrier methods, hormonal implants and tubal ligation. Women with high-risk cardiac conditions should avoid combined hormonal contraception due to an increased risk of hypertension and stroke (70). Amongst women with AHF, intrauterine devices are the most appropriate contraceptive method (66). Following HT, there are additional concerns including drug-drug interactions with immunosuppressive medications (65). The ISHLT recommends against intrauterine devices due to an increased risk of expulsion in nulliparous women and concerns about increased risks of infection after HT (71). However, the Center for Disease Control and Prevention supports the use of intrauterine devices in women with complex medical conditions including solid organ transplant (72).

### Menopause

There is accumulating evidence that the menopausal transition influences HF risk (73). Menopause is associated with metabolic derangements, inflammation and lipid abnormalities that promote an adverse cardiovascular risk profile and HF development (14). It is unclear if the increase in HF risk after menopause is predominantly due to hormonal changes that occur with the menopausal transition, or result from a higher prevalence of risk factors that occur with biologic aging (14). Even in the absence of biochemical markers of myocardial injury, early menopause is independently associated with HF development (74). The type of menopause also influences HF risk. When compared to those with natural menopause, women with surgical menopause have worse cardiovascular risk profiles prior to menopause, and the adverse changes in LV structure and function seen among them may be explained by their pre-surgical risk profile (75). Cardiovascular risk factors such as obesity and hypertension which affect HF progression influence both age at natural menopause and indications for surgical menopause (75). For instance, uncontrolled hypertension could trigger hospitalization, progression to advanced disease, and poor outcomes (76) in postmenopausal women with HF.

## Barriers To Therapies For Women With Advanced Heart Failure

**Figure 3:**
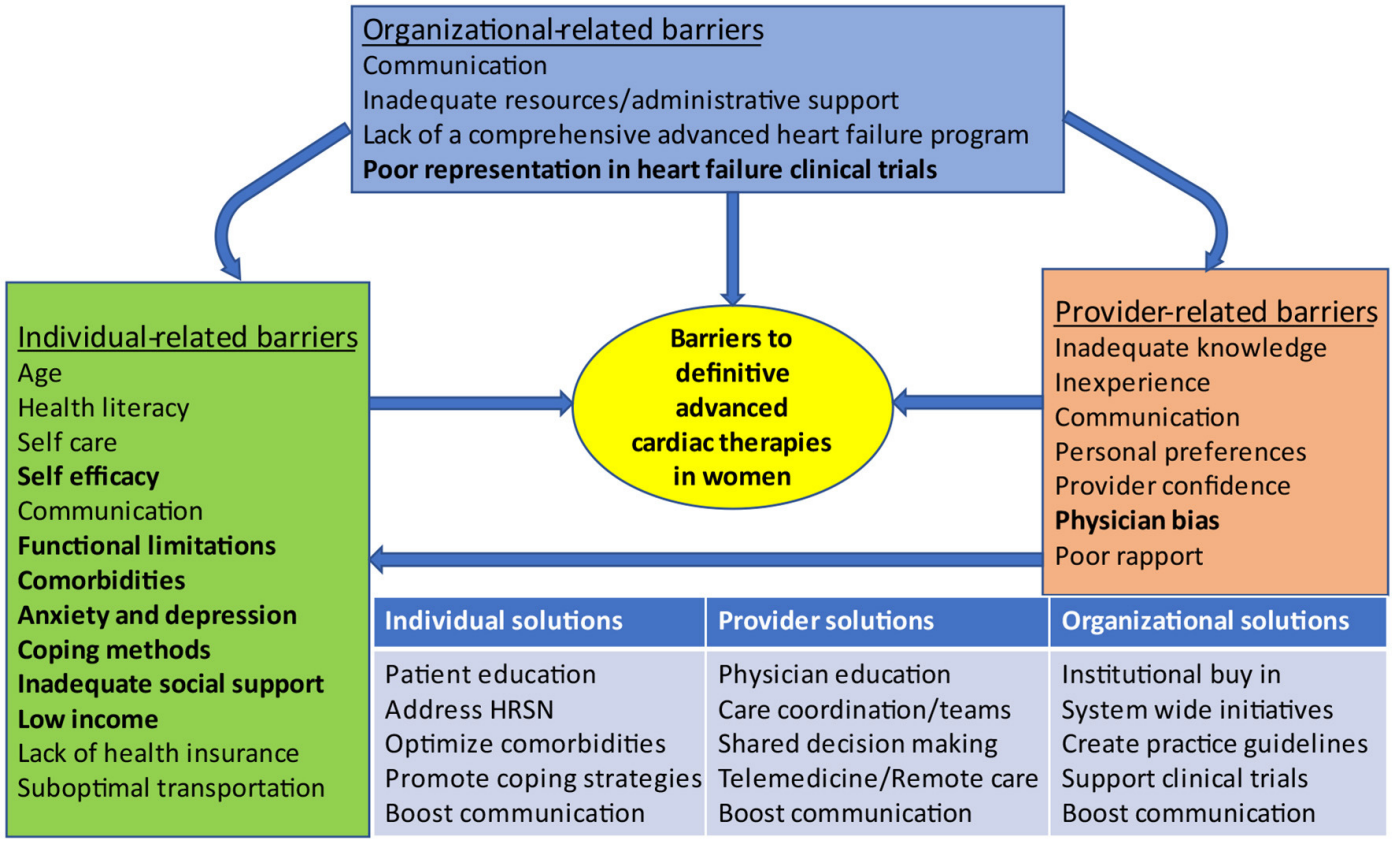
Barriers to advanced heart failure therapies in women and proposed solutions. Organizational-related barriers can influence individual-related and provider-related barriers. Provider-related barriers can influence individual-related barriers. Factors that are more common in women appear in bold. HRSN, Health related social needs.

Barriers to AHF care prevail at the individual, provider and organizational levels (77) with well recognized gender differences (78) (Figure 3). Social determinants of health, including lack of health insurance, low income, and inadequate social support, are more prevalent among women, and contribute to physician bias in decision making, which promotes worse outcomes, delayed referrals and decreased access to advanced therapies for women (2). Self-care, an integral component of HF management is greatly affected by self-efficacy and functional status in women (79). Depression also negatively impacts self-care and is present in as many as 35% of HF patients (80). Depression, social isolation and poor support systems are recognized barriers to HF self-care in women (78). A woman's caregiving responsibilities may hinder her from seeking care due to conflicting priorities (38). Actual or perceived inadequacy in social support is an important barrier to equitable allocation of advanced therapies in women (10). African American women may be appraised more harshly (10), and are often perceived as having more financial and social challenges when compared to White patients and men (38). Strategies targeting barriers to advanced therapies in women must also be implemented at the individual, provider and organizational levels (Figure 3). Organizational policies especially those guiding the implementation of an integrated AHF program can influence individual and provider factors that affect candidacy for AHF therapies.

## Conclusion And Future Directions

HF progression is influenced by sex-specific risk factors which should be considered when evaluating women for advanced therapies. The management of AHF in women is highly complex and requires effective integration of conventional treatments, advanced therapies and palliative care to achieve optimal outcomes. There are persisting inequities in allocation of advanced therapies, and women are less likely to be referred for a HT or LVAD evaluation despite facing a higher risk of AHF-related mortality. Future studies should address areas of equipoise in the management of AHF among women (Table 2). Women should be given equal opportunities as men for inclusion in clinical trials on AHF.

**Table 2:** Future areas of study in the management of advanced heart failure in women.

**Areas of equipoise in the management of advanced heart failure in women**	**Potential areas of future research**
Hemodynamic instability	Identify sex-specific cutoffs that indicate hemodynamic compromise in men and women
Biomarker derangements	Explore the validity of sex-specific cutoffs for advanced heart failure prognostication
Exercise capacity	Investigate sex specific cut-offs in 6-min walk test, exercise duration and functional capacity
Frailty assessment	Explore the optimal strategy for frailty assessment among women with advanced heart failure
Waitlisting prior to heart transplant	Identify the optimal waitlist strategy for female patients in the pre-transplant period
Chronic inotrope use	Impact of chronic inotropes on sex-based clinical outcomes
Palliative care	Explore a sex-specific approach to the use of palliative care interventions
Temporary mechanical circulatory support	Evaluate sex-specific differences in the utilization and outcomes of temporary mechanical circulatory support
Anticoagulation strategy in LVAD	Explore optimal anticoagulation strategies in male and female LVAD patients
Referral for advanced therapies	Evaluate the role of a sex-specific risk stratification strategy in referrals for advanced cardiac therapies
Allocation of advanced therapies	Evaluate impact of interventions aimed at reducing inequities in allocation of advanced cardiac therapies

*LVAD, left ventricular assist devices*.

## Author Contributions

IE proposed the study. IE, ED, EAH, VR, MK, AB, MB, MM, RR, AH, and CG contributed to design of the study and drafting the initial manuscript. IE, ED, EAH, VR, FB, TD, and SH contributed to editing and revising of the manuscript for intellectual content. EMH, FB, TD, and SH provided critical feedback. All authors approved the final version of the manuscript.

## Conflict of Interest

The authors declare that the research was conducted in the absence of any commercial or financial relationships that could be construed as a potential conflict of interest.

## Publisher's Note

All claims expressed in this article are solely those of the authors and do not necessarily represent those of their affiliated organizations, or those of the publisher, the editors and the reviewers. Any product that may be evaluated in this article, or claim that may be made by its manufacturer, is not guaranteed or endorsed by the publisher.

## Abbreviations

AHF: advanced heart failure
HT: heart transplant
INTERMACS: Interagency Registry for Mechanically Assisted Circulatory Support
ISHLT: International Society of Heart and Lung Transplantation
LVAD: left ventricular assist device
MOMENTUM 3: Multicenter Study of MagLev Technology in Patients Undergoing Mechanical Circulatory Support Therapy with HeartMate 3
PPCM: peripartum cardiomyopathy
REVIVAL: Registry Evaluation for Vital Information on Ventricular Assist Devices in Ambulatory Life
SHOCK: SHould we emergently revascularize Occluded Coronaries for cardiogenic shock
TMCS: temporary mechanical circulatory support.
